# Supplementary material for: Trends and future burden of major digestive system cancers in Jiangsu Province, China, 2010–2030
Source: Front Oncol. 2026 Sep 9;16:1902194. doi: 10.3389/fonc.2026.1902194 (PMC13597408; doi:10.3389/fonc.2026.1902194)
Supplement: Supplementary file 1 [file Table1.docx]

| **Year** | **Overall** | **Urban** | **Rural** |
| --- | --- | --- | --- |
| **2010** | 27 | 9 | 18 |
| **2011** | 34 | 10 | 24 |
| **2012** | 34 | 10 | 24 |
| **2013** | 34 | 10 | 24 |
| **2014** | 32 | 9 | 23 |
| **2015** | 35 | 10 | 25 |
| **2016** | 41 | 10 | 31 |
| **2017** | 43 | 11 | 32 |
| **2018** | 48 | 19 | 29 |
| **2019** | 54 | 23 | 31 |
| **2020** | 69 | 30 | 39 |
| **2021** | 74 | 33 | 41 |

**Supplement Table 1.** The number of qualified registry sites in Jiangsu from 2010 to 2021

**Supplement Table 2.** The primary quality control indicators of the Jiangsu cancer registration process from 2010 to 2021

| **Year** | **M/I** | **DCO (%)** | **MV (%)** |
| --- | --- | --- | --- |
| **2010** | 0.69 | 1.21 | 68.00 |
| **2011-2013** | 0.68 | 0.97 | 67.55 |
| **2014** | 0.67 | 0.82 | 68.20 |
| **2015** | 0.67 | 0.61 | 68.70 |
| **2016** | 0.66 | 0.71 | 68.70 |
| **2017** | 0.64 | 1.07 | 70.04 |
| **2018** | 0.63 | 0.92 | 69.79 |
| **2019** | 0.61 | 0.72 | 70.41 |
| **2020** | 0.60 | 0.93 | 71.44 |
| **2021** | 0.57 | 0.62 | 73.55 |

**Supplement Table 3.** Cancer-specific quality control indicators for the digestive system cancers from 2010 to 2021

| **Year** | **ICD-10** | **Cancer Name** | **Overall** | | | **Urban** | | | **Rural** | | |
| --- | --- | --- | --- | --- | --- | --- | --- | --- | --- | --- | --- |
|  |  |  | **M/I** | **MV (%)** | **DCO (%)** | **M/I** | **MV (%)** | **DCO (%)** | **M/I** | **MV (%)** | **DCO (%)** |
| 2010 | C15 | Esophageal cancer | 0.74 | 81.02 | 0.86 | 0.75 | 77.77 | 0.86 | 0.74 | 83.01 | 0.87 |
| 2010 | C16 | Gastric cancer | 0.74 | 84.42 | 0.89 | 0.74 | 81.80 | 1.01 | 0.74 | 87.06 | 0.77 |
| 2010 | C18-C21 | Colorectal cancer | 0.53 | 83.47 | 0.48 | 0.49 | 80.57 | 0.64 | 0.57 | 87.07 | 0.29 |
| 2010 | C22 | Liver cancer | 0.93 | 34.40 | 2.33 | 0.95 | 40.80 | 3.79 | 0.92 | 30.44 | 1.43 |
| 2011-2013 | C15 | Esophageal cancer | 0.76 | 81.83 | 0.80 | 0.77 | 78.85 | 1.41 | 0.75 | 83.36 | 0.48 |
| 2011-2013 | C16 | Gastric cancer | 0.73 | 82.43 | 0.88 | 0.71 | 79.75 | 1.29 | 0.74 | 84.85 | 0.52 |
| 2011-2013 | C18-C21 | Colorectal cancer | 0.48 | 81.35 | 0.70 | 0.45 | 78.82 | 0.98 | 0.52 | 84.51 | 0.36 |
| 2011-2013 | C22 | Liver cancer | 0.93 | 31.86 | 1.43 | 0.91 | 41.45 | 2.01 | 0.94 | 25.24 | 1.03 |
| 2014 | C15 | Esophageal cancer | 0.77 | 84.52 | 0.92 | 0.80 | 81.79 | 0.63 | 0.76 | 85.71 | 1.05 |
| 2014 | C16 | Gastric cancer | 0.74 | 86.05 | 0.65 | 0.75 | 84.68 | 0.38 | 0.73 | 87.12 | 0.87 |
| 2014 | C18-C21 | Colorectal cancer | 0.48 | 83.89 | 0.47 | 0.46 | 81.56 | 0.28 | 0.52 | 86.44 | 0.68 |
| 2014 | C22 | Liver cancer | 0.91 | 31.58 | 1.31 | 0.92 | 37.34 | 1.08 | 0.90 | 28.24 | 1.44 |
| 2015 | C15 | Esophageal cancer | 0.78 | 81.87 | 0.56 | 0.78 | 79.99 | 0.93 | 0.78 | 82.70 | 0.41 |
| 2015 | C16 | Gastric cancer | 0.75 | 82.12 | 0.52 | 0.76 | 79.59 | 0.57 | 0.75 | 83.95 | 0.48 |
| 2015 | C18-C21 | Colorectal cancer | 0.49 | 83.47 | 0.35 | 0.49 | 81.06 | 0.49 | 0.49 | 85.56 | 0.24 |
| 2015 | C22 | Liver cancer | 0.92 | 31.18 | 1.05 | 0.96 | 32.98 | 1.23 | 0.90 | 30.29 | 0.96 |
| 2016 | C15 | Esophageal cancer | 0.81 | 83.27 | 0.67 | 0.84 | 80.85 | 1.33 | 0.81 | 84.19 | 0.42 |
| 2016 | C16 | Gastric cancer | 0.75 | 83.04 | 0.54 | 0.73 | 80.32 | 0.75 | 0.76 | 84.59 | 0.41 |
| 2016 | C18-C21 | Colorectal cancer | 0.47 | 83.45 | 0.45 | 0.46 | 81.09 | 0.79 | 0.48 | 85.09 | 0.21 |
| 2016 | C22 | Liver cancer | 0.90 | 33.52 | 1.17 | 0.93 | 34.29 | 2.24 | 0.89 | 33.18 | 0.68 |
| 2017 | C15 | Esophageal cancer | 0.84 | 81.20 | 0.91 | 0.82 | 79.93 | 1.40 | 0.85 | 81.70 | 0.72 |
| 2017 | C16 | Gastric cancer | 0.74 | 81.58 | 0.85 | 0.70 | 80.95 | 1.61 | 0.76 | 81.95 | 0.40 |
| 2017 | C18-C21 | Colorectal cancer | 0.47 | 84.19 | 0.76 | 0.46 | 83.57 | 1.36 | 0.48 | 84.67 | 0.30 |
| 2017 | C22 | Liver cancer | 0.93 | 36.33 | 1.76 | 0.93 | 37.51 | 3.41 | 0.92 | 35.72 | 0.91 |
| 2018 | C15 | Esophageal cancer | 0.86 | 79.28 | 0.81 | 0.86 | 77.25 | 1.48 | 0.86 | 80.49 | 0.41 |
| 2018 | C16 | Gastric cancer | 0.74 | 81.26 | 0.90 | 0.73 | 79.08 | 1.47 | 0.75 | 83.05 | 0.42 |
| 2018 | C18-C21 | Colorectal cancer | 0.48 | 82.14 | 0.84 | 0.48 | 79.54 | 1.41 | 0.48 | 84.73 | 0.26 |
| 2018 | C22 | Liver cancer | 0.90 | 36.31 | 1.45 | 0.90 | 33.39 | 2.25 | 0.90 | 38.51 | 0.86 |
| 2019 | C15 | Esophageal cancer | 0.86 | 78.48 | 0.68 | 0.85 | 76.34 | 1.23 | 0.87 | 79.93 | 0.31 |
| 2019 | C16 | Gastric cancer | 0.74 | 79.39 | 0.60 | 0.73 | 77.55 | 0.95 | 0.75 | 81.09 | 0.26 |
| 2019 | C18-C21 | Colorectal cancer | 0.48 | 82.14 | 0.67 | 0.48 | 81.22 | 1.00 | 0.47 | 83.12 | 0.32 |
| 2019 | C22 | Liver cancer | 0.94 | 38.84 | 1.18 | 0.95 | 35.59 | 1.84 | 0.92 | 41.42 | 0.66 |
| 2020 | C15 | Esophageal cancer | 0.85 | 78.04 | 1.24 | 0.85 | 75.76 | 1.46 | 0.85 | 79.43 | 1.10 |
| 2020 | C16 | Gastric cancer | 0.77 | 79.21 | 1.02 | 0.75 | 78.24 | 1.10 | 0.78 | 80.00 | 0.95 |
| 2020 | C18-C21 | Colorectal cancer | 0.46 | 82.67 | 0.63 | 0.46 | 82.68 | 0.71 | 0.46 | 82.66 | 0.56 |
| 2020 | C22 | Liver cancer | 0.92 | 37.15 | 1.86 | 0.92 | 36.53 | 2.29 | 0.92 | 37.60 | 1.55 |
| 2021 | C15 | Esophageal cancer | 0.85 | 78.83 | 0.75 | 0.85 | 76.76 | 0.93 | 0.85 | 80.18 | 0.64 |
| 2021 | C16 | Gastric cancer | 0.77 | 79.80 | 0.63 | 0.74 | 78.80 | 0.74 | 0.80 | 80.68 | 0.53 |
| 2021 | C18-C21 | Colorectal cancer | 0.46 | 83.54 | 0.43 | 0.46 | 82.87 | 0.48 | 0.46 | 84.24 | 0.38 |
| 2021 | C22 | Liver cancer | 0.95 | 38.10 | 1.31 | 0.97 | 38.44 | 1.33 | 0.93 | 37.86 | 1.30 |

**Supplement Table 4.** Imputed annual age-standardized rates for the combined 2011-2013 reporting period.

| Outcome | Stratum | Cancer code | Cancer | Reported combined 2011-2013 ASR | Imputed 2011 ASR | Imputed 2012 ASR | Imputed 2013 ASR | Mean of imputed 2011-2013 ASR |
| --- | --- | --- | --- | --- | --- | --- | --- | --- |
| Incidence | Overall | C15 | Esophageal cancer | 24.17 | 23.99 | 24.17 | 24.35 | 24.17 |
| Incidence | Overall | C16 | Gastric cancer | 27.76 | 27.21 | 27.76 | 28.31 | 27.76 |
| Incidence | Overall | C18 | Colon cancer | 6.57 | 5.848 | 6.57 | 7.293 | 6.57 |
| Incidence | Overall | C19-20 | Rectal cancer | 7.43 | 6.82 | 7.43 | 8.04 | 7.43 |
| Incidence | Overall | C22 | Liver cancer | 20.07 | 19.558 | 20.07 | 20.582 | 20.07 |
| Incidence | Male | C15 | Esophageal cancer | 33.44 | 33.233 | 33.44 | 33.648 | 33.44 |
| Incidence | Male | C16 | Gastric cancer | 40.02 | 39.51 | 40.02 | 40.53 | 40.02 |
| Incidence | Male | C18 | Colon cancer | 7.65 | 6.79 | 7.65 | 8.51 | 7.65 |
| Incidence | Male | C19-20 | Rectal cancer | 8.85 | 8.092 | 8.85 | 9.607 | 8.85 |
| Incidence | Male | C22 | Liver cancer | 30.07 | 29.425 | 30.07 | 30.715 | 30.07 |
| Incidence | Female | C15 | Esophageal cancer | 15.18 | 15.053 | 15.18 | 15.307 | 15.18 |
| Incidence | Female | C16 | Gastric cancer | 15.95 | 15.4 | 15.95 | 16.5 | 15.95 |
| Incidence | Female | C18 | Colon cancer | 5.54 | 4.957 | 5.54 | 6.122 | 5.54 |
| Incidence | Female | C19-20 | Rectal cancer | 6.07 | 5.615 | 6.07 | 6.525 | 6.07 |
| Incidence | Female | C22 | Liver cancer | 10.14 | 9.76 | 10.14 | 10.52 | 10.14 |
| Incidence | Urban | C15 | Esophageal cancer | 18.32 | 18.933 | 18.32 | 17.707 | 18.32 |
| Incidence | Urban | C16 | Gastric cancer | 29.51 | 29.4 | 29.51 | 29.62 | 29.51 |
| Incidence | Urban | C18 | Colon cancer | 8.97 | 7.9 | 8.97 | 10.04 | 8.97 |
| Incidence | Urban | C19-20 | Rectal cancer | 8.31 | 7.678 | 8.31 | 8.943 | 8.31 |
| Incidence | Urban | C22 | Liver cancer | 18.04 | 17.483 | 18.04 | 18.598 | 18.04 |
| Incidence | Rural | C15 | Esophageal cancer | 28.9 | 28.207 | 28.9 | 29.593 | 28.9 |
| Incidence | Rural | C16 | Gastric cancer | 26.35 | 25.427 | 26.35 | 27.273 | 26.35 |
| Incidence | Rural | C18 | Colon cancer | 4.63 | 4.12 | 4.63 | 5.14 | 4.63 |
| Incidence | Rural | C19-20 | Rectal cancer | 6.72 | 6.11 | 6.72 | 7.33 | 6.72 |
| Incidence | Rural | C22 | Liver cancer | 21.76 | 21.372 | 21.76 | 22.148 | 21.76 |
| Mortality | Overall | C15 | Esophageal cancer | 17.59 | 17.225 | 17.59 | 17.955 | 17.59 |
| Mortality | Overall | C16 | Gastric cancer | 19.26 | 18.845 | 19.26 | 19.675 | 19.26 |
| Mortality | Overall | C18 | Colon cancer | 2.63 | 2.35 | 2.63 | 2.91 | 2.63 |
| Mortality | Overall | C19-20 | Rectal cancer | 3.64 | 3.42 | 3.64 | 3.86 | 3.64 |
| Mortality | Overall | C22 | Liver cancer | 18.24 | 17.875 | 18.24 | 18.605 | 18.24 |
| Mortality | Male | C15 | Esophageal cancer | 24.65 | 24.065 | 24.65 | 25.235 | 24.65 |
| Mortality | Male | C16 | Gastric cancer | 27.74 | 27.235 | 27.74 | 28.245 | 27.74 |
| Mortality | Male | C18 | Colon cancer | 3.07 | 2.78 | 3.07 | 3.36 | 3.07 |
| Mortality | Male | C19-20 | Rectal cancer | 4.43 | 4.175 | 4.43 | 4.685 | 4.43 |
| Mortality | Male | C22 | Liver cancer | 27.48 | 26.99 | 27.48 | 27.97 | 27.48 |
| Mortality | Female | C15 | Esophageal cancer | 10.9 | 10.773 | 10.9 | 11.027 | 10.9 |
| Mortality | Female | C16 | Gastric cancer | 11.33 | 11.037 | 11.33 | 11.623 | 11.33 |
| Mortality | Female | C18 | Colon cancer | 2.24 | 1.972 | 2.24 | 2.507 | 2.24 |
| Mortality | Female | C19-20 | Rectal cancer | 2.91 | 2.735 | 2.91 | 3.085 | 2.91 |
| Mortality | Female | C22 | Liver cancer | 9.1 | 8.865 | 9.1 | 9.335 | 9.1 |
| Mortality | Urban | C15 | Esophageal cancer | 13.66 | 13.898 | 13.66 | 13.422 | 13.66 |
| Mortality | Urban | C16 | Gastric cancer | 19.93 | 19.77 | 19.93 | 20.09 | 19.93 |
| Mortality | Urban | C18 | Colon cancer | 3.38 | 2.962 | 3.38 | 3.797 | 3.38 |
| Mortality | Urban | C19-20 | Rectal cancer | 3.89 | 3.712 | 3.89 | 4.067 | 3.89 |
| Mortality | Urban | C22 | Liver cancer | 15.98 | 15.602 | 15.98 | 16.358 | 15.98 |
| Mortality | Rural | C15 | Esophageal cancer | 20.74 | 19.977 | 20.74 | 21.503 | 20.74 |
| Mortality | Rural | C16 | Gastric cancer | 18.72 | 18.105 | 18.72 | 19.335 | 18.72 |
| Mortality | Rural | C18 | Colon cancer | 2.03 | 1.837 | 2.03 | 2.223 | 2.03 |
| Mortality | Rural | C19-20 | Rectal cancer | 3.44 | 3.183 | 3.44 | 3.697 | 3.44 |
| Mortality | Rural | C22 | Liver cancer | 20.12 | 19.85 | 20.12 | 20.39 | 20.12 |

**Supplement Figure 1.** Surveyed population in Jiangsu from 2010 to 2021. (A) Overall; (B) By sex; (C) By residence.

**Supplement Table 5** Segment-specific APC estimates of incidence trends of five major digestive system cancers from 2010 to 2021

| **Stratum** | **Cancer Name** | **Model** | **Segment** | **Segment Start** | **Segment End** | **APC** | **APC_LCL** | **APC_UCL** | **Significant** | **P Value** |
| --- | --- | --- | --- | --- | --- | --- | --- | --- | --- | --- |
| Overall | Esophageal cancer | 2 | 0 | 2010 | 2012 | 10.7663 | 2.1584 | 19.3556 | 1 | 0.017197 |
| Overall | Esophageal cancer | 2 | 1 | 2012 | 2017 | -8.8813 | -11.7391 | -7.4667 | 1 | 0 |
| Overall | Esophageal cancer | 2 | 2 | 2017 | 2021 | -5.9565 | -7.6188 | -2.2713 | 1 | 0.0012 |
| Overall | Gastric cancer | 1 | 0 | 2010 | 2012 | 10.9626 | -1.2049 | 25.3862 | 0 | 0.095981 |
| Overall | Gastric cancer | 1 | 1 | 2012 | 2021 | -4.8262 | -6.7585 | -3.7813 | 1 | 0.002 |
| Overall | Colon cancer | 1 | 0 | 2010 | 2012 | 24.9674 | 10.6138 | 39.4982 | 1 | 0 |
| Overall | Colon cancer | 1 | 1 | 2012 | 2021 | 4.7109 | 3.6306 | 5.6584 | 1 | 0.003599 |
| Overall | Rectal cancer | 1 | 0 | 2010 | 2012 | 20.3242 | 8.269 | 32.1278 | 1 | 0 |
| Overall | Rectal cancer | 1 | 1 | 2012 | 2021 | 0.3525 | -0.4968 | 1.1594 | 0 | 0.312338 |
| Overall | Liver cancer | 1 | 0 | 2010 | 2012 | 12.1537 | 4.0503 | 19.773 | 1 | 0.004799 |
| Overall | Liver cancer | 1 | 1 | 2012 | 2021 | -6.0215 | -6.7286 | -5.3772 | 1 | 0 |
| Male | Esophageal cancer | 2 | 0 | 2010 | 2012 | 10.1085 | 1.9921 | 17.8148 | 1 | 0.013597 |
| Male | Esophageal cancer | 2 | 1 | 2012 | 2016 | -8.7613 | -11.1095 | -6.9638 | 1 | 0 |
| Male | Esophageal cancer | 2 | 2 | 2016 | 2021 | -5.4562 | -6.5113 | -2.231 | 1 | 0.0008 |
| Male | Gastric cancer | 1 | 0 | 2010 | 2012 | 9.4875 | -1.839 | 21.5968 | 0 | 0.128374 |
| Male | Gastric cancer | 1 | 1 | 2012 | 2021 | -4.7902 | -6.6837 | -3.8164 | 1 | 0.0012 |
| Male | Colon cancer | 1 | 0 | 2010 | 2012 | 25.4626 | 8.7995 | 47.3798 | 1 | 0 |
| Male | Colon cancer | 1 | 1 | 2012 | 2021 | 5.269 | 3.4065 | 6.4951 | 1 | 0.019596 |
| Male | Rectal cancer | 1 | 0 | 2010 | 2012 | 21.2722 | 7.2156 | 37.941 | 1 | 0 |
| Male | Rectal cancer | 1 | 1 | 2012 | 2021 | 1.0948 | -0.0816 | 2.0607 | 0 | 0.065987 |
| Male | Liver cancer | 1 | 0 | 2010 | 2012 | 11.2178 | 2.1652 | 20.3957 | 1 | 0.015197 |
| Male | Liver cancer | 1 | 1 | 2012 | 2021 | -6.0451 | -6.92 | -5.316 | 1 | 0 |
| Female | Esophageal cancer | 1 | 0 | 2010 | 2012 | 10.7597 | -3.446 | 27.1102 | 0 | 0.171566 |
| Female | Esophageal cancer | 1 | 1 | 2012 | 2021 | -9.1489 | -10.8361 | -8.01 | 1 | 0 |
| Female | Gastric cancer | 1 | 0 | 2010 | 2012 | 13.5284 | 1.4447 | 24.2602 | 1 | 0.023195 |
| Female | Gastric cancer | 1 | 1 | 2012 | 2021 | -4.7158 | -5.9089 | -3.7962 | 1 | 0 |
| Female | Colon cancer | 1 | 0 | 2010 | 2012 | 23.9208 | 7.8315 | 45.033 | 1 | 0 |
| Female | Colon cancer | 1 | 1 | 2012 | 2021 | 3.9729 | 1.8935 | 5.2168 | 1 | 0.025995 |
| Female | Rectal cancer | 1 | 0 | 2010 | 2012 | 18.4536 | 7.2146 | 28.7178 | 1 | 0 |
| Female | Rectal cancer | 1 | 1 | 2012 | 2021 | -0.755 | -1.5673 | 0.0314 | 0 | 0.059988 |
| Female | Liver cancer | 1 | 0 | 2010 | 2012 | 14.9299 | 3.746 | 25.6769 | 1 | 0.004799 |
| Female | Liver cancer | 1 | 1 | 2012 | 2021 | -5.7778 | -6.7144 | -4.9201 | 1 | 0 |
| Urban | Esophageal cancer | 2 | 0 | 2010 | 2012 | 0.6613 | -4.1649 | 7.66 | 0 | 0.987403 |
| Urban | Esophageal cancer | 2 | 1 | 2012 | 2017 | -6.9896 | -10.1216 | -5.569 | 1 | 0 |
| Urban | Esophageal cancer | 2 | 2 | 2017 | 2021 | -3.378 | -4.9415 | 0.0988 | 0 | 0.054789 |
| Urban | Gastric cancer | 1 | 0 | 2010 | 2012 | 6.4939 | -1.7937 | 14.3172 | 0 | 0.161568 |
| Urban | Gastric cancer | 1 | 1 | 2012 | 2021 | -5.1497 | -6.2455 | -4.4251 | 1 | 0 |
| Urban | Colon cancer | 1 | 0 | 2010 | 2012 | 25.9945 | 7.1663 | 47.8746 | 1 | 0 |
| Urban | Colon cancer | 1 | 1 | 2012 | 2021 | 2.1654 | 0.1985 | 3.5554 | 1 | 0.045991 |
| Urban | Rectal cancer | 2 | 0 | 2010 | 2012 | 19.6607 | 7.5042 | 35.8336 | 1 | 0 |
| Urban | Rectal cancer | 2 | 1 | 2012 | 2018 | -1.2745 | -5.6022 | 0.2828 | 0 | 0.087582 |
| Urban | Rectal cancer | 2 | 2 | 2018 | 2021 | 1.8965 | -1.9961 | 6.5324 | 0 | 0.117177 |
| Urban | Liver cancer | 1 | 0 | 2010 | 2012 | 12.6698 | -0.6824 | 26.9314 | 0 | 0.071986 |
| Urban | Liver cancer | 1 | 1 | 2012 | 2021 | -5.3149 | -7.1921 | -4.1662 | 1 | 0.0008 |
| Rural | Esophageal cancer | 2 | 0 | 2010 | 2012 | 17.7028 | 5.9661 | 26.686 | 1 | 0.0004 |
| Rural | Esophageal cancer | 2 | 1 | 2012 | 2016 | -11.267 | -14.025 | -8.9932 | 1 | 0 |
| Rural | Esophageal cancer | 2 | 2 | 2016 | 2021 | -6.8855 | -8.1054 | -3.1563 | 1 | 0 |
| Rural | Gastric cancer | 1 | 0 | 2010 | 2012 | 16.1061 | -1.3541 | 41.1908 | 0 | 0.09878 |
| Rural | Gastric cancer | 1 | 1 | 2012 | 2021 | -4.7042 | -10.7648 | -3.1953 | 1 | 0.006799 |
| Rural | Colon cancer | 1 | 0 | 2010 | 2016 | 13.7405 | 10.408 | 50.1003 | 1 | 0 |
| Rural | Colon cancer | 1 | 1 | 2016 | 2021 | 4.9026 | -2.2572 | 7.6994 | 0 | 0.084783 |
| Rural | Rectal cancer | 1 | 0 | 2010 | 2012 | 24.7283 | 8.1545 | 42.3183 | 1 | 0 |
| Rural | Rectal cancer | 1 | 1 | 2012 | 2021 | 0.5744 | -0.6708 | 1.6369 | 0 | 0.29994 |
| Rural | Liver cancer | 1 | 0 | 2010 | 2012 | 10.4463 | 2.4112 | 17.8809 | 1 | 0.009998 |
| Rural | Liver cancer | 1 | 1 | 2012 | 2021 | -6.3385 | -7.0706 | -5.7115 | 1 | 0 |

**Supplement Table 6** Segment-specific APC estimates of mortality trends of five major digestive system cancers from 2010 to 2021

| **Stratum** | **Cancer Name** | **Model** | **Segment** | **Segment Start** | **Segment End** | **APC** | **APC_LCL** | **APC_UCL** | **Significant** | **P Value** |
| --- | --- | --- | --- | --- | --- | --- | --- | --- | --- | --- |
| Overall | Esophageal cancer | 1 | 0 | 2010 | 2012 | 10.7961 | 2.6871 | 17.2165 | 1 | 0.005999 |
| Overall | Esophageal cancer | 1 | 1 | 2012 | 2021 | -6.2829 | -6.8809 | -5.7026 | 1 | 0 |
| Overall | Gastric cancer | 1 | 0 | 2010 | 2012 | 11.3906 | 0.1363 | 23.5324 | 1 | 0.045991 |
| Overall | Gastric cancer | 1 | 1 | 2012 | 2021 | -4.8394 | -6.0325 | -3.9519 | 1 | 0 |
| Overall | Colon cancer | 1 | 0 | 2010 | 2012 | 24.3795 | 6.9118 | 46.4761 | 1 | 0 |
| Overall | Colon cancer | 1 | 1 | 2012 | 2021 | 3.7321 | 1.491 | 5.0183 | 1 | 0.031994 |
| Overall | Rectal cancer | 1 | 0 | 2010 | 2012 | 16.852 | 4.6759 | 28.419 | 1 | 0 |
| Overall | Rectal cancer | 1 | 1 | 2012 | 2021 | -0.8477 | -1.8549 | 0.0159 | 0 | 0.054789 |
| Overall | Liver cancer | 1 | 0 | 2010 | 2012 | 10.9024 | 2.0918 | 18.8318 | 1 | 0.011598 |
| Overall | Liver cancer | 1 | 1 | 2012 | 2021 | -6.0677 | -6.8282 | -5.3897 | 1 | 0 |
| Male | Esophageal cancer | 1 | 0 | 2010 | 2012 | 10.5563 | 2.0261 | 17.3373 | 1 | 0.012398 |
| Male | Esophageal cancer | 1 | 1 | 2012 | 2021 | -5.5433 | -6.2003 | -4.9418 | 1 | 0 |
| Male | Gastric cancer | 1 | 0 | 2010 | 2012 | 10.3742 | -0.5873 | 22.2432 | 0 | 0.065187 |
| Male | Gastric cancer | 1 | 1 | 2012 | 2021 | -4.549 | -5.7981 | -3.6809 | 1 | 0.0008 |
| Male | Colon cancer | 1 | 0 | 2010 | 2019 | 6.4152 | 5.3995 | 19.8688 | 1 | 0.003199 |
| Male | Colon cancer | 1 | 1 | 2019 | 2021 | -0.1487 | -4.8165 | 5.0573 | 0 | 0.784643 |
| Male | Rectal cancer | 1 | 0 | 2010 | 2012 | 16.756 | 3.7393 | 30.1935 | 1 | 0.0004 |
| Male | Rectal cancer | 1 | 1 | 2012 | 2021 | 0.1274 | -1.304 | 1.1198 | 0 | 0.869426 |
| Male | Liver cancer | 1 | 0 | 2010 | 2012 | 10.6111 | 0.9749 | 20.1443 | 1 | 0.029994 |
| Male | Liver cancer | 1 | 1 | 2012 | 2021 | -6.1405 | -7.095 | -5.3552 | 1 | 0 |
| Female | Esophageal cancer | 1 | 0 | 2010 | 2012 | 10.5029 | 2.5324 | 16.818 | 1 | 0.015597 |
| Female | Esophageal cancer | 1 | 1 | 2012 | 2021 | -7.8474 | -8.4373 | -7.2813 | 1 | 0 |
| Female | Gastric cancer | 1 | 0 | 2010 | 2012 | 12.5429 | -1.9137 | 32.1757 | 0 | 0.122775 |
| Female | Gastric cancer | 1 | 1 | 2012 | 2021 | -5.2792 | -8.531 | -4.0442 | 1 | 0.003999 |
| Female | Colon cancer | 1 | 0 | 2010 | 2012 | 28.5349 | 8.8916 | 55.5481 | 1 | 0 |
| Female | Colon cancer | 1 | 1 | 2012 | 2021 | 2.7743 | 1.0978 | 4.0834 | 1 | 0.023595 |
| Female | Rectal cancer | 1 | 0 | 2010 | 2012 | 16.1072 | 5.2252 | 22.7676 | 1 | 0 |
| Female | Rectal cancer | 1 | 1 | 2012 | 2021 | -2.3981 | -3.0353 | -1.7536 | 1 | 0 |
| Female | Liver cancer | 1 | 0 | 2010 | 2012 | 11.4064 | 3.4496 | 18.8066 | 1 | 0.005199 |
| Female | Liver cancer | 1 | 1 | 2012 | 2021 | -5.6324 | -6.2603 | -5.0257 | 1 | 0 |
| Urban | Esophageal cancer | 0 | 0 | 2010 | 2021 | -4.0101 | -4.5363 | -3.3224 | 1 | 0 |
| Urban | Gastric cancer | 1 | 0 | 2010 | 2013 | 2.6764 | -1.9684 | 12.2242 | 0 | 0.333933 |
| Urban | Gastric cancer | 1 | 1 | 2013 | 2021 | -5.7817 | -6.8427 | -4.9784 | 1 | 0 |
| Urban | Colon cancer | 1 | 0 | 2010 | 2013 | 16.8131 | 8.8961 | 37.2201 | 1 | 0 |
| Urban | Colon cancer | 1 | 1 | 2013 | 2021 | 1.8163 | 0.6788 | 2.9097 | 1 | 0.011598 |
| Urban | Rectal cancer | 1 | 0 | 2010 | 2012 | 13.9958 | 1.7117 | 27.3439 | 1 | 0.006799 |
| Urban | Rectal cancer | 1 | 1 | 2012 | 2021 | -1.2321 | -3.1489 | -0.1812 | 1 | 0.021996 |
| Urban | Liver cancer | 1 | 0 | 2010 | 2012 | 11.1498 | 0.8128 | 21.8306 | 1 | 0.027994 |
| Urban | Liver cancer | 1 | 1 | 2012 | 2021 | -5.1969 | -6.1867 | -4.3606 | 1 | 0 |
| Rural | Esophageal cancer | 1 | 0 | 2010 | 2012 | 15.4282 | 2.3657 | 26.5544 | 1 | 0.013197 |
| Rural | Esophageal cancer | 1 | 1 | 2012 | 2021 | -7.0736 | -8.0887 | -6.1666 | 1 | 0 |
| Rural | Gastric cancer | 1 | 0 | 2010 | 2012 | 15.2989 | -0.9763 | 37.9153 | 0 | 0.088782 |
| Rural | Gastric cancer | 1 | 1 | 2012 | 2021 | -4.3245 | -7.8648 | -2.8862 | 1 | 0.006799 |
| Rural | Colon cancer | 1 | 0 | 2010 | 2018 | 8.8063 | 7.1791 | 31.5852 | 1 | 0.0008 |
| Rural | Colon cancer | 1 | 1 | 2018 | 2021 | 0.2142 | -9.0961 | 5.1916 | 0 | 0.85223 |
| Rural | Rectal cancer | 1 | 0 | 2010 | 2012 | 20.4069 | 7.1291 | 34.2507 | 1 | 0 |
| Rural | Rectal cancer | 1 | 1 | 2012 | 2021 | -0.6533 | -1.6306 | 0.229 | 0 | 0.141172 |
| Rural | Liver cancer | 1 | 0 | 2010 | 2012 | 9.3871 | 0.2776 | 16.2769 | 1 | 0.045191 |
| Rural | Liver cancer | 1 | 1 | 2012 | 2021 | -6.529 | -7.3734 | -5.8444 | 1 | 0 |

**Supplement Table 7** Sensitivity analysis for handling the combined 2011-2013 reporting interval

| Outcome | Stratum | Cancer code | Cancer | AAPC retained midpoint (%) | AAPC primary split-imputed (%) | 2026 forecast retained midpoint | 2026 forecast primary split-imputed | Relative difference in 2026 forecast (%) | Interpretation |
| --- | --- | --- | --- | --- | --- | --- | --- | --- | --- |
| Mortality | Overall | C15 | Esophageal cancer | -3.697 | -3.386 | 7.214 | 7.193 | -0.29 | Stable (absolute difference <=10%) |
| Mortality | Overall | C16 | Gastric cancer | -2.332 | -2.075 | 9.748 | 9.715 | -0.34 | Stable (absolute difference <=10%) |
| Mortality | Overall | C18 | Colon cancer | 6.752 | 7.213 | 4.461 | 4.468 | 0.15 | Stable (absolute difference <=10%) |
| Mortality | Overall | C19-C20 | Rectal cancer | 1.594 | 2.158 | 3.31 | 3.412 | 3.08 | Stable (absolute difference <=10%) |
| Mortality | Overall | C22 | Liver cancer | -3.495 | -3.188 | 7.796 | 7.788 | -0.1 | Stable (absolute difference <=10%) |
| Mortality | Male | C15 | Esophageal cancer | -3.154 | -2.801 | 11.248 | 11.188 | -0.53 | Stable (absolute difference <=10%) |
| Mortality | Male | C16 | Gastric cancer | -2.431 | -1.994 | 14.356 | 14.531 | 1.22 | Stable (absolute difference <=10%) |
| Mortality | Male | C18 | Colon cancer | 5.548 | 5.19 | 4.375 | 4.447 | 1.65 | Stable (absolute difference <=10%) |
| Mortality | Male | C19-C20 | Rectal cancer | 2.548 | 2.964 | 4.529 | 4.69 | 3.55 | Stable (absolute difference <=10%) |
| Mortality | Male | C22 | Liver cancer | -3.616 | -3.296 | 11.599 | 11.553 | -0.4 | Stable (absolute difference <=10%) |
| Mortality | Female | C15 | Esophageal cancer | -5.04 | -4.754 | 3.549 | 3.549 | 0 | Stable (absolute difference <=10%) |
| Mortality | Female | C16 | Gastric cancer | -2.534 | -2.263 | 5.373 | 5.368 | -0.09 | Stable (absolute difference <=10%) |
| Mortality | Female | C18 | Colon cancer | 6.499 | 7.04 | 3.433 | 3.451 | 0.52 | Stable (absolute difference <=10%) |
| Mortality | Female | C19-C20 | Rectal cancer | 0.386 | 0.732 | 2.216 | 2.241 | 1.13 | Stable (absolute difference <=10%) |
| Mortality | Female | C22 | Liver cancer | -3.045 | -2.741 | 4.168 | 4.183 | 0.35 | Stable (absolute difference <=10%) |
| Mortality | Urban | C15 | Esophageal cancer | -3.835 | -4.01 | 7.328 | 7.261 | -0.91 | Stable (absolute difference <=10%) |
| Mortality | Urban | C16 | Gastric cancer | -3.555 | -3.547 | 9.338 | 9.34 | 0.02 | Stable (absolute difference <=10%) |
| Mortality | Urban | C18 | Colon cancer | 5.593 | 5.704 | 4.929 | 4.935 | 0.12 | Stable (absolute difference <=10%) |
| Mortality | Urban | C19-C20 | Rectal cancer | 0.962 | 1.377 | 3.383 | 3.506 | 3.64 | Stable (absolute difference <=10%) |
| Mortality | Urban | C22 | Liver cancer | -2.723 | -2.415 | 7.635 | 7.658 | 0.3 | Stable (absolute difference <=10%) |
| Mortality | Rural | C15 | Esophageal cancer | -3.756 | -3.337 | 7.546 | 7.477 | -0.91 | Stable (absolute difference <=10%) |
| Mortality | Rural | C16 | Gastric cancer | -1.765 | -1.024 | 9.434 | 9.901 | 4.95 | Stable (absolute difference <=10%) |
| Mortality | Rural | C18 | Colon cancer | 6.898 | 6.393 | 3.07 | 3.113 | 1.42 | Stable (absolute difference <=10%) |
| Mortality | Rural | C19-C20 | Rectal cancer | 2.478 | 2.881 | 3.272 | 3.31 | 1.16 | Stable (absolute difference <=10%) |
| Mortality | Rural | C22 | Liver cancer | -4.09 | -3.818 | 8.069 | 7.984 | -1.06 | Stable (absolute difference <=10%) |
| Incidence | Overall | C15 | Esophageal cancer | -5.048 | -4.497 | 8.571 | 9.195 | 7.28 | Stable (absolute difference <=10%) |
| Incidence | Overall | C16 | Gastric cancer | -2.374 | -2.133 | 13.972 | 13.908 | -0.46 | Stable (absolute difference <=10%) |
| Incidence | Overall | C18 | Colon cancer | 7.14 | 8.133 | 12.297 | 12.89 | 4.82 | Stable (absolute difference <=10%) |
| Incidence | Overall | C19-C20 | Rectal cancer | 3.32 | 3.719 | 8.131 | 8.122 | -0.11 | Stable (absolute difference <=10%) |
| Incidence | Overall | C22 | Liver cancer | -3.29 | -2.951 | 8.588 | 8.628 | 0.46 | Stable (absolute difference <=10%) |
| Incidence | Male | C15 | Esophageal cancer | -4.631 | -4.049 | 13.277 | 13.922 | 4.86 | Stable (absolute difference <=10%) |
| Incidence | Male | C16 | Gastric cancer | -2.914 | -2.34 | 19.287 | 19.911 | 3.23 | Stable (absolute difference <=10%) |
| Incidence | Male | C18 | Colon cancer | 7.64 | 8.682 | 15.203 | 15.758 | 3.65 | Stable (absolute difference <=10%) |
| Incidence | Male | C19-C20 | Rectal cancer | 4.053 | 4.496 | 10.661 | 10.644 | -0.16 | Stable (absolute difference <=10%) |
| Incidence | Male | C22 | Liver cancer | -3.459 | -3.119 | 12.777 | 12.798 | 0.16 | Stable (absolute difference <=10%) |
| Incidence | Female | C15 | Esophageal cancer | -6.13 | -5.816 | 4.209 | 4.221 | 0.3 | Stable (absolute difference <=10%) |
| Incidence | Female | C16 | Gastric cancer | -1.925 | -1.632 | 8.276 | 8.31 | 0.41 | Stable (absolute difference <=10%) |
| Incidence | Female | C18 | Colon cancer | 6.433 | 7.344 | 9.564 | 10.158 | 6.21 | Stable (absolute difference <=10%) |
| Incidence | Female | C19-C20 | Rectal cancer | 2.125 | 2.49 | 5.745 | 5.738 | -0.12 | Stable (absolute difference <=10%) |
| Incidence | Female | C22 | Liver cancer | -2.678 | -2.312 | 4.538 | 4.589 | 1.11 | Stable (absolute difference <=10%) |
| Incidence | Urban | C15 | Esophageal cancer | -4.734 | -4.327 | 8.781 | 9.423 | 7.32 | Stable (absolute difference <=10%) |
| Incidence | Urban | C16 | Gastric cancer | -3.317 | -3.132 | 14.451 | 14.456 | 0.03 | Stable (absolute difference <=10%) |
| Incidence | Urban | C18 | Colon cancer | 5.607 | 6.135 | 13.376 | 13.557 | 1.35 | Stable (absolute difference <=10%) |
| Incidence | Urban | C19-C20 | Rectal cancer | 2.444 | 3.124 | 8.606 | 9.403 | 9.27 | Stable (absolute difference <=10%) |
| Incidence | Urban | C22 | Liver cancer | -2.611 | -2.273 | 8.28 | 8.234 | -0.55 | Stable (absolute difference <=10%) |
| Incidence | Rural | C15 | Esophageal cancer | -5.286 | -4.521 | 8.867 | 9.478 | 6.89 | Stable (absolute difference <=10%) |
| Incidence | Rural | C16 | Gastric cancer | -2.052 | -1.22 | 12.607 | 13.352 | 5.91 | Stable (absolute difference <=10%) |
| Incidence | Rural | C18 | Colon cancer | 10.396 | 9.634 | 10.999 | 11.04 | 0.37 | Stable (absolute difference <=10%) |
| Incidence | Rural | C19-C20 | Rectal cancer | 3.811 | 4.588 | 7.474 | 7.738 | 3.54 | Stable (absolute difference <=10%) |
| Incidence | Rural | C22 | Liver cancer | -3.797 | -3.489 | 9.02 | 9.046 | 0.29 | Stable (absolute difference <=10%) |
